# Inadvertent temporary transvenous pacing of the left ventricle: an underreported complication—a case report

**DOI:** 10.1093/ehjcr/ytaf299

**Published:** 2025-06-25

**Authors:** Girish Pathangey, Mahmoud H Abdelnabi, Ramzi Ibrahim, Hemalatha Narayanasamy, Dan Sorajja

**Affiliations:** Department of Cardiovascular Disease, Mayo Clinic Arizona, 5777 E. Mayo Blvd, USA, Phoenix, AZ 85054; Department of Cardiovascular Disease, Mayo Clinic Arizona, 5777 E. Mayo Blvd, USA, Phoenix, AZ 85054; Department of Cardiovascular Disease, Mayo Clinic Arizona, 5777 E. Mayo Blvd, USA, Phoenix, AZ 85054; Department of Cardiovascular Disease, Mayo Clinic Arizona, 5777 E. Mayo Blvd, USA, Phoenix, AZ 85054; Department of Cardiovascular Disease, Mayo Clinic Arizona, 5777 E. Mayo Blvd, USA, Phoenix, AZ 85054

**Keywords:** Complication, Electrophysiology, Cardiac pacemaker, Multimodal imaging, Temporary transvenous pacing, Atrioventricular block, Case report

## Abstract

**Background:**

Inadvertent temporary transvenous pacing (TTVP) of left ventricle (LV) is rare and likely underreported, posing significant embolic risk. We present a case of TTVP with atypical trajectory identified on computed tomography (CT) imaging.

**Summary:**

A 62-year-old male with metastatic renal cell carcinoma on chemotherapy and bifascicular block presented with symptomatic bradycardia and non–ST-elevation myocardial infarction (NSTEMI), raising concerns for complete heart block on ECG. Intermittent asystole occurred during TTVP placement with appropriate capture. Post-procedure, ECG showed ventricular-paced rhythm with pseudo-right bundle branch block, and chest X-ray suggested lead placement in right ventricle; however, suboptimal echocardiography limited lead visualization. Coronary angiography revealed non-obstructive coronary artery disease. Positron emission tomography–computed tomography (PET-CT), performed to evaluate immune checkpoint inhibitor myocarditis, incidentally noted TTVP wire entering right subclavian artery and traversing to LV. Patient underwent TTVP removal, endovascular repair, and pacemaker implantation; however, course was unfortunately complicated by embolic stroke and haemorrhagic conversion.

**Discussion:**

Early detection and management of lead malposition remain critical to minimizing complications. Management strategies for inadvertent lead malposition (ILM) depend on duration of implantation, clinical presentation, and associated risks. This case highlights importance of recognizing this high-risk complication, preventive strategies, and evidence-based management. While existing data primarily focus on ILM in permanent devices, further research is needed to elucidate incidence, predictors, and outcomes of ILM in TTVP, particularly in resource-limited settings.

Learning points

- Inadvertent temporary transvenous pacing of the left ventricle is a rare but high-risk complication.
- Early detection and management of lead malposition remain challenging yet critical to minimizing associated complications.
- Limited available data highlight the need for further research to optimize strategies for prevention, identification, and management.

## Introduction

Inadvertent lead malposition (ILM) in the left ventricle (LV) is a rare but severe complication associated with thromboembolism and valvular damage.^1^ While the incidence is underreported, one cohort observed ILM in 0.34% of 1764 patients undergoing cardiac device implantation, though temporary transvenous pacing (TTVP) was not specifically evaluated.^2^ We report a case of inadvertent TTVP in the LV, with atypical lead trajectory identified on CT imaging.

## Summary figure

**Figure:**
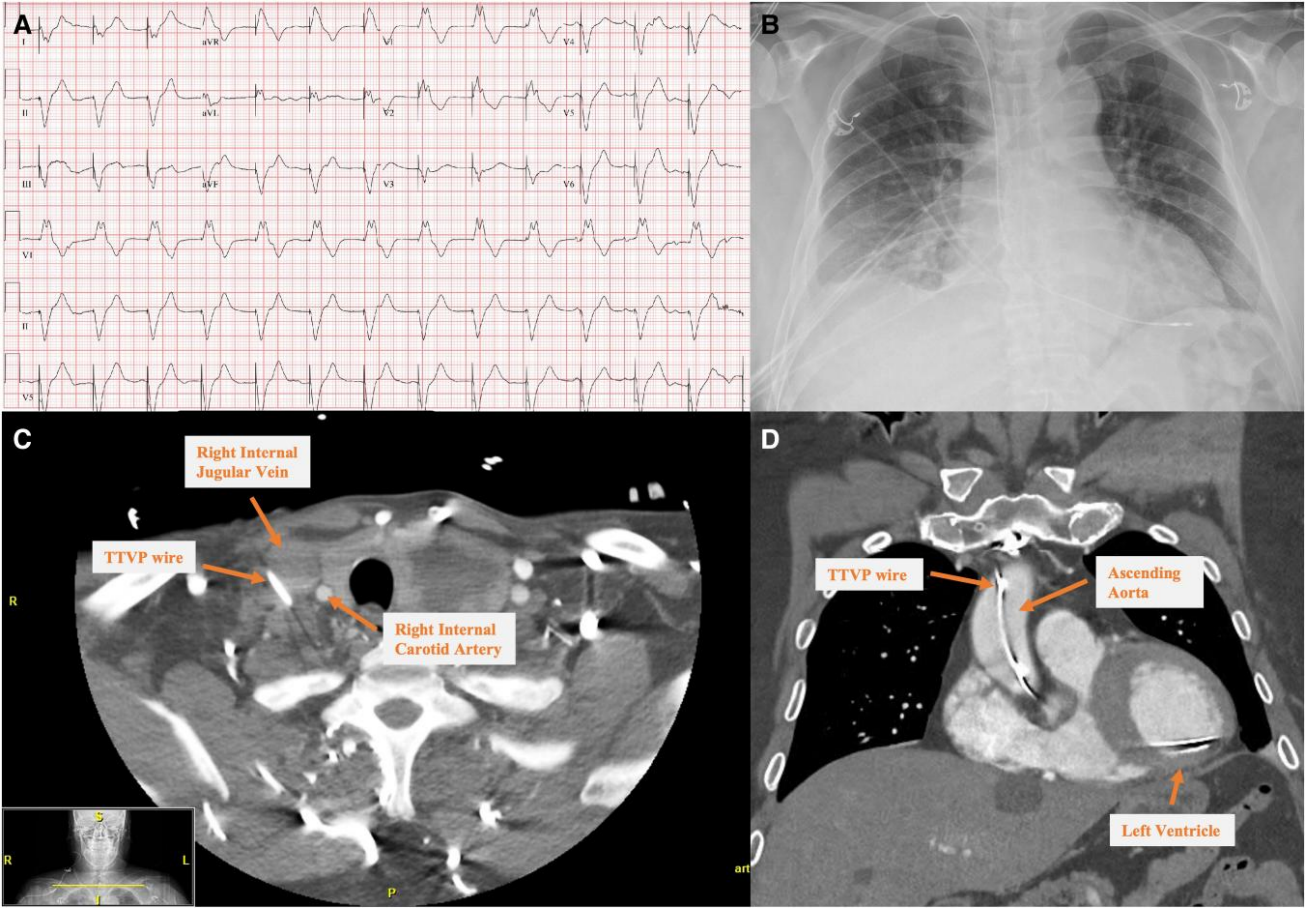
(*A*) Post-procedure ECG showing ventricular-paced rhythm with a pseudo-right bundle branch block. (*B*) Anteroposterior chest X-ray depicting wire placement on the right side of the vertebral body, suggesting trajectory into the right heart. (*C, D*) Temporary pacing wire lateral to the right internal jugular vein, incidentally located in the right subclavian/brachiocephalic artery and ascending aorta, with the lead positioned in the left ventricle.

## Case presentation

**Figure 1:**
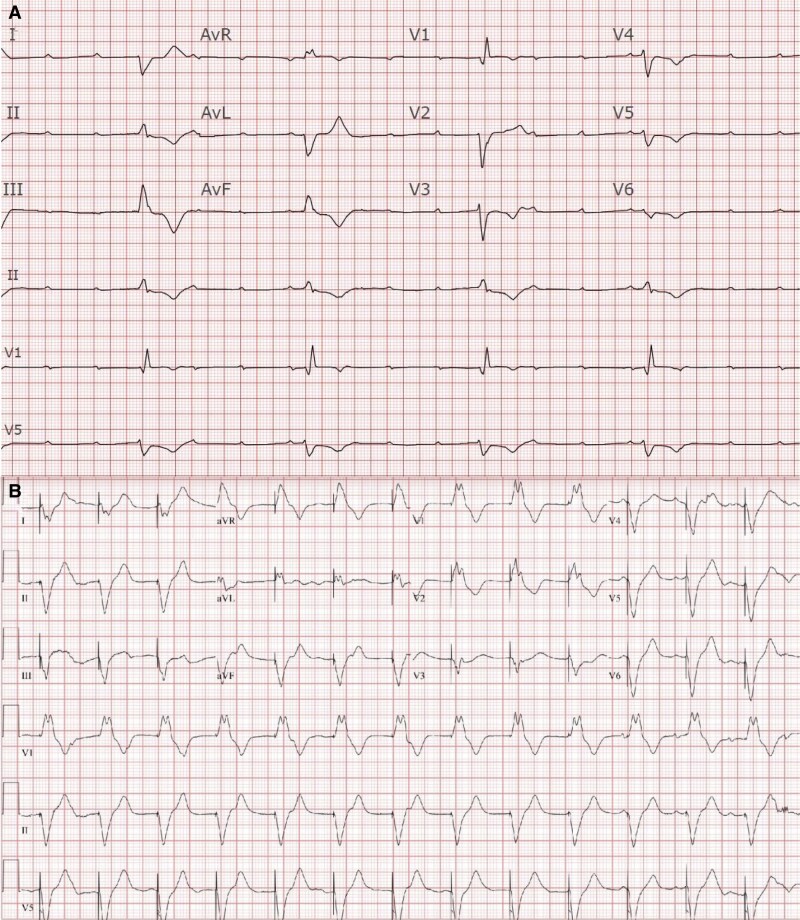
(*A*) Admission ECG: complete heart block with right bundle branch escape rhythm. (*C*) Post-procedure ECG: ventricular-paced rhythm and pseudo-right bundle branch block.

A 62-year-old man with metastatic renal cell carcinoma and bifascicular block, not on atrioventricular (AV) nodal agents, presented with 48 h worsening dizziness, dyspnoea, and fatigue. On admission, he was bradycardic (heart rate 27 b.p.m.) with regular S1/S2, no murmurs, jugular venous distention, or edema; he was diaphoretic, lightheaded, and short of breath but haemodynamically stable, with symptoms refractory to atropine. Labs revealed elevated high-sensitivity troponin T at 1114 ng/L (reference ≤15 ng/L) and N-terminal pro–B-type natriuretic peptide at 2543 pg/mL (reference ≤88 pg/mL). Electrocardiogram (ECG) demonstrated complete heart block (CHB) with right bundle branch escape (*Figure 1A*), and transthoracic echocardiogram (TTE) showed left ventricular ejection fraction (LVEF) 65% without wall motion abnormalities.

Due to symptomatic CHB refractory to medication, patient underwent TTVP following guidelines and shared decision-making with patient and family. The modified Seldinger technique was used to cannulate the right internal jugular vein for central venous sheath insertion, complicated by intermittent asystole preventing adequate assessment of venous vs. arterial access. The temporary screw-in pacing lead was advanced to presumed apical right ventricular (RV) septum with capture threshold of 0.7 mA, and then connected to an externalized pulse generator set to VVI mode 80 b.p.m. with output 10 mA.

**Figure 2:**
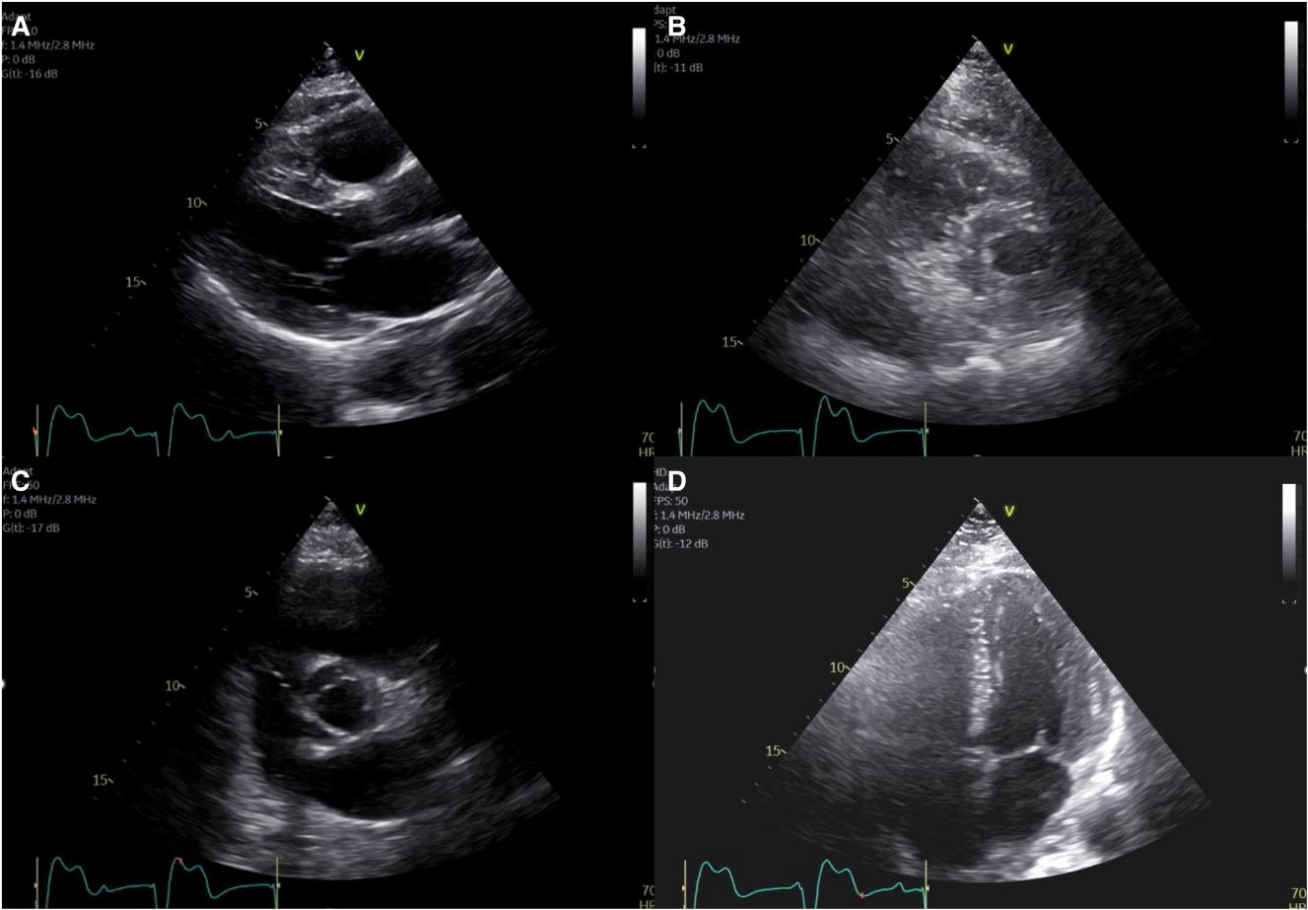
(*A*) Parasternal long axis view (PLAX) view: EF 55%, mildly enlarged left atrium (LA), and mild apical septal/inferior hypokinesis, likely due to temporary transvenous pacing. (*B*) parasternal short axis view (PSAX) view. (*C*) PSAX view of aortic valve: lead not visualized. (*D*) Apical four-chamber view.

**Figure 3:**
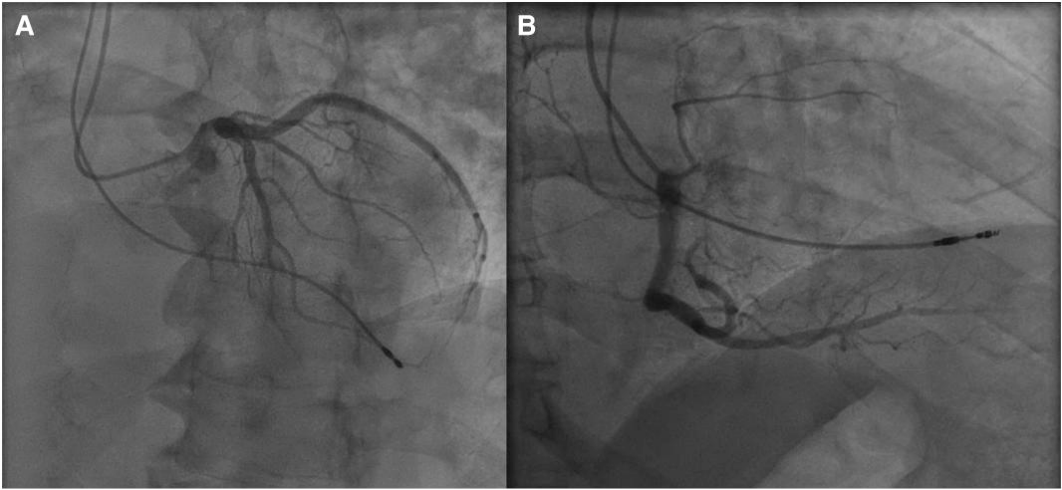
(*A*) Coronary angiogram left anterior oblique cranial view of left coronary system. (*B*) right anterior oblique straight view of right coronary system.

Post-procedure ECG showed ventricular-paced rhythm with pseudo-right bundle branch block (RBBB), and anteroposterior (AP) chest X-ray suggested apical RV lead placement (Central Illustration B). Limited TTE revealed LVEF 55%, but assessment was limited by paced rhythm and lead was not visualized (*Figure 2*). Coronary angiography demonstrated non-obstructive coronary artery disease with 30% ostial stenosis in small first diagonal artery (*Figure 3*).

Oncology managed suspected immune checkpoint inhibitor (ICI) myocarditis with high-dose steroids. Despite treatment response evidenced by declining troponin and inflammatory markers, underlying CHB persisted. Following discussion—considering recent diagnosis of Stage IV metastatic renal cell carcinoma (RCC), preserved functional status prior to presentation, and hospice ineligibility—the patient opted for palliative permanent pacemaker (PPM) implantation. Proptosis prompted empiric pyridostigmine for possible ICI-related myasthenia gravis. Head CT revealed posterior right occipital transcortical hypoattenuation (suggestive developing infarct vs. metastases) and multifocal right cerebellar hypoattenuation consistent with chronic infarcts.

**Figure 4:**
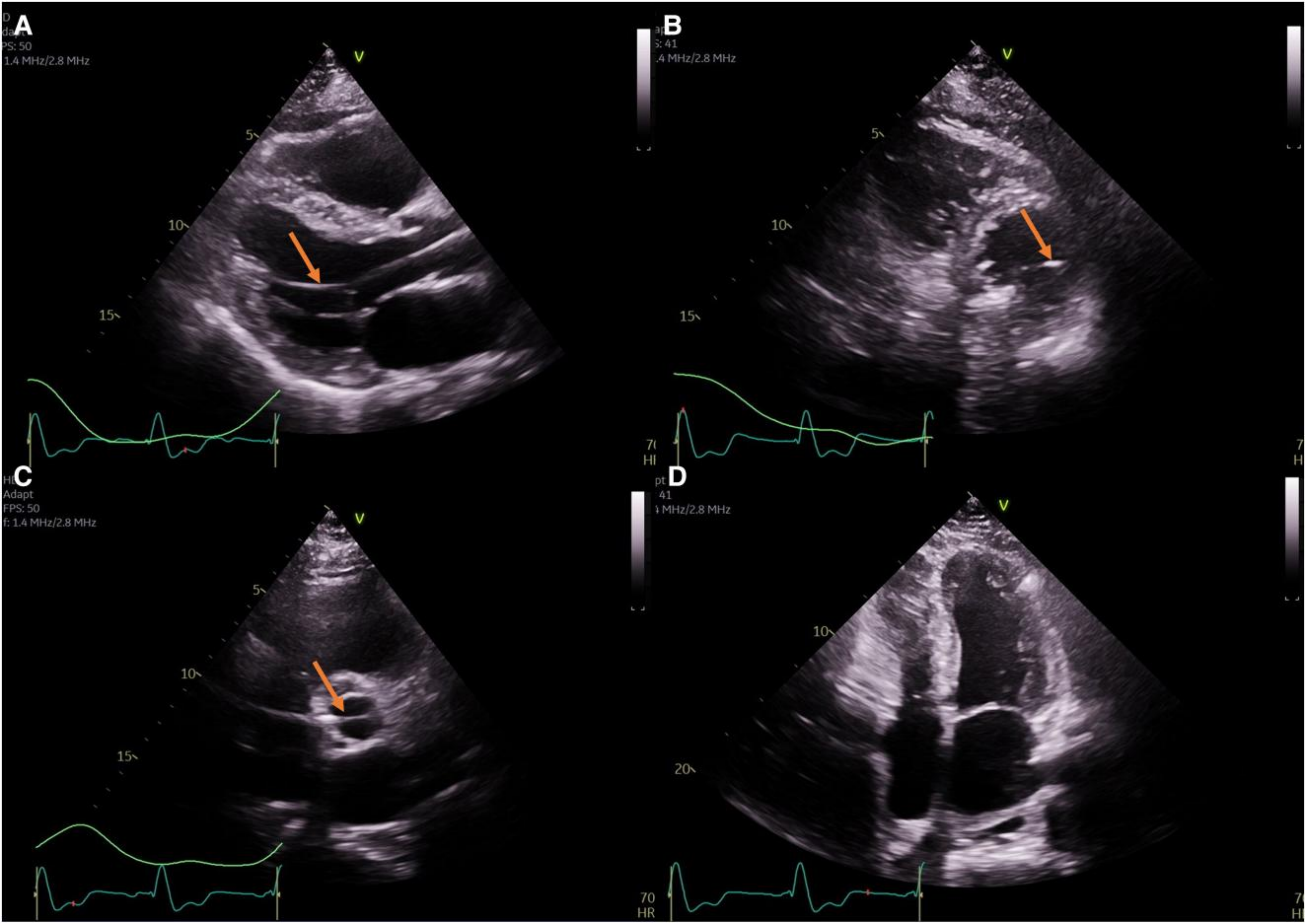
(*A*) PLAX view: lead in the ascending aorta, crossing the aortic valve, positioned in the lateral left ventricle. (*B*) PSAX view: lead in lateral inferior left ventricle. (*C*) PSAX view: lead crossing the aortic valve. (*D*) Apical four-chamber view: lead not visualized.

**Figure 5:**
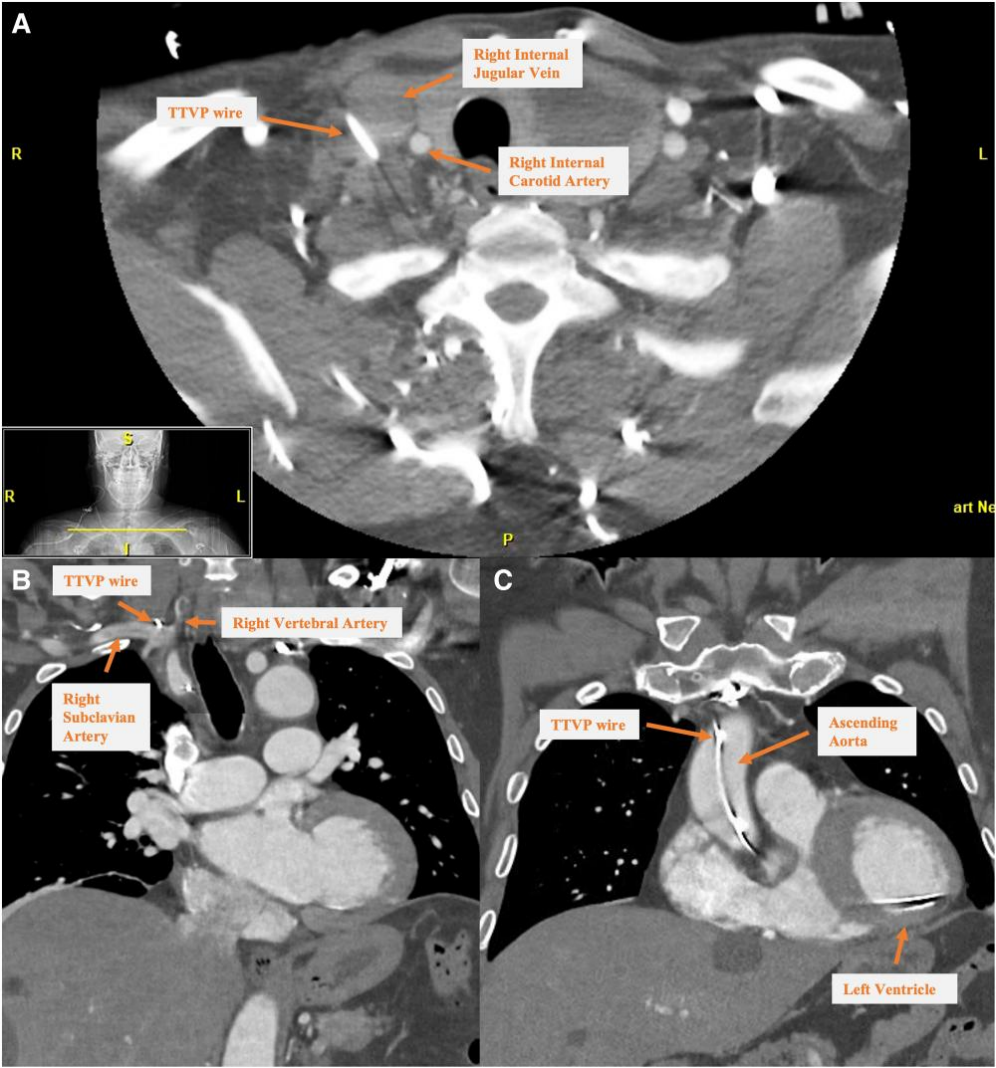
(*A*) CT: temporary transvenous pacing wire lateral to right IJ vein. (*B*, *C*) Wire entering superior vena cava, subclavian artery, brachiocephalic artery, and ascending aorta, with lead in left ventricle.

PET-CT evaluating ICI myocarditis revealed diffuse myocardial fluorodeoxyglucose uptake and incidentally identified TTVP lead in LV. Repeat TTE confirmed the lead crossing the aortic valve into the LV (*Figure 4*), and CT showed entry via the right subclavian artery, traversing the brachiocephalic artery and aorta into the LV (*Figure 5*).

Vascular surgery was consulted for TTVP removal and endovascular right subclavian artery repair. Under transoesophageal echocardiography guidance, the lead was retracted from LV to the ascending aorta. Right brachial artery exposure and angiography confirmed lead entry into the superior right subclavian artery. A Gore VBX stent-graft was deployed, the TTVP lead was removed, and PPM was implanted. Post-procedure imaging confirmed no extravasation and preserved perfusion.

5.5 h post-anaesthesia, patient developed left-sided weakness. Computed tomography revealed early ischaemia in the right middle cerebral artery (MCA) territory, with occlusion of the right internal carotid artery and proximal MCA. The patient was ineligible for thrombolytics, thereby underwent emergent mechanical thrombectomy via right femoral artery access using a Solitaire stent retriever and intra-arterial tissue plasminogen activator. Despite intervention, post-procedure CT showed evolving right MCA infarction with haemorrhagic conversion and worsening mass effect. Following multidisciplinary goals-of-care discussions, the family opted for comfort care, and subsequently patient died 48 h post stroke.

## Discussion

Inadvertent TTVP placement was first reported in 1969,^3^ with subsequent reports limited to isolated cases and small series. Compared with PPM implantation, TTVP carries higher risk profile. Here, we review contributing factors, preventive strategies, and evidence-based management of this complication.

The patient underwent TTVP placement following 2018 ACC/AHA/HRS guidelines (Class of Recommendation [COR] IIa; Level of Evidence [LOE] B-NR) for symptomatic CHB refractory to medication.^4^ Given atropine resistance, CHB was suspected to be infranodal in origin, and thus unlikely responsive to isoproterenol, dopamine, dobutamine, or epinephrine, which primarily enhance AV nodal conduction.^4^ Infusion therapy with these dromotropic agents was not pursued due to haemodynamic stability and lower controllability compared to TTVP, pending evaluation for reversible causes such as ICI before definitive PPM consideration.

The 2021 ESC guidelines recommend fluoroscopy during lead placement (COR IIa, LOE C) to confirm positioning and prevent complications,^5^ and expert consensus further emphasizes the importance of fluoroscopic checks in AP and lateral views to improve placement precision.^6^ In this case, routine use of orthogonal projections (LAO/RAO) did not reveal wire misplacement toward the left spine or buckling at the aortic valve. Intermittent asystole may have masked arterial pulsations, delaying recognition.

Postoperative ECG demonstrating RBBB pattern should prompt suspicion of ILM; however, pseudo-RBBB can occur during RV apical pacing in 16% of cases, whereas true LV ILM is rarer (<2%).^7,8^ Pseudo-RBBB may result from variable V1/V2 lead positioning, altering QRS patterns from RBBB to left bundle branch block during RV pacing.^1^ Here, pseudo-RBBB was attributed to RV lead placement near the apical septum, consistent with expected right paramedian course on AP chest X-ray (CXR), though the absence of a lateral view may have limited ILM detection. Expert consensus recommends both AP and lateral views for precise lead placement,^9^ but lateral views are not routinely obtained during TTVP placement at our institution.

2021 ESC guidelines recommend fluoroscopy during lead placement (COR IIa, LOE C) to confirm positioning and prevent complications, and expert consensus further emphasizes the importance of fluoroscopic checks in AP and lateral views to improve placement precision.^6^ In this case, routine orthogonal projections (LAO/RAO) did not reveal wire misplacement toward the left spine or buckling at the aortic valve. Intermittent asystole may have masked arterial pulsations, delaying recognition.

^5^Coronary catheterization may theoretically engage an ILM traversing the aorta; however, this phenomenon is not consistently observed despite utilization of a Tiger catheter, known for its propensity to flip. Differentiation between catheter and lead on 2D fluoroscopy remains technically challenging, but the lead here demonstrated a more medial trajectory (*Figure 5*).

A similar ILM course was described by Yoshimoto *et al*.,^10^ with the lead puncturing the subclavian artery and entering the LV, remaining undetected for 2 months. Recognition of ILM is often delayed and detected postoperatively through imaging or ECG, particularly in asymptomatic patients. Factors including scoliosis, abnormal anatomy, operator inexperience, and emergency or out-of-hours procedures increase ILM risk.^2,5^ Early recognition and management, including lead extraction or long-term anticoagulation, are recommended to reduce long-term complications (COR I, LOE B).^9,11^ However, existing data pertain to PPM, with limited evidence for TTVP.

Management depends on implantation duration, clinical presentation, and associated complications. Leads implanted <3 months undergo percutaneous extraction with simple traction, though arterial complications may necessitate balloon tamponade, stent-graft placement, or surgery. Extraction is generally avoided in chronic cases (>1 year) unless infection present—favouring long-term anticoagulation (international normalized ratio 2.5–3.5)—due to significant risks including valvular damage and embolization (up to 40%), although cerebral embolic protection devices recently employed to mitigate risks.^1,5,11,12^

Inadvertent lead malposition is a preventable complication; this case highlights procedural checkpoints and emphasizes adherence to established best practices during transvenous pacing. Routine ultrasound-guided vascular access, fluoroscopic advancement of guidewire below diaphragm into inferior vena cava, and orthogonal fluoroscopy facilitate confirmation of venous placement and prevent arterial puncture or left-sided lead placement via patent foramen ovale/septal defects. Foley *et al*.^1^ emphasize intraoperative ILM detection methods, including intracardiac electrograms, arterial pulsation upon sheath insertion, and recognition of atypical guidewire trajectories. Postprocedural assessment includes monitoring pacing-induced RBBB and CXR, with further evaluation by echocardiography, angiography, or CT as indicated. Structured intra- and postoperative checklists with multidisciplinary team involvement are recommended to identify aberrant lead positions and reduce associated risks (see Supplementary material online, *Table*).

## Conclusion

Inadvertent lead malposition is a rare but serious complication, necessitating careful review of intra- and postoperative diagnostics for early identification. While current data largely pertains to PPM, further research is needed to define incidence, predictors and outcomes in TTVP, particularly in resource-limited critical care or emergency settings.

## Supplementary Material

ytaf299_Supplementary_Data

## Data Availability

The data underlying this article are available in the article and in its online Supplementary material.

## Lead author biography

Dr Girish Pathangey is a cardiology fellow at Mayo Clinic Arizona. He completed his residency at Dartmouth Health and earned his medical degree from Oakland University William Beaumont. Prior to medical school, he graduated from Arizona State University with a degree in Biomedical Engineering.
