# Supplementary material for: Inadvertent temporary transvenous pacing of the left ventricle: an underreported complication—a case report
Source: Eur Heart J Case Rep. 2025 Jun 25;9(7):ytaf299. doi: 10.1093/ehjcr/ytaf299 (PMC12255227; doi:10.1093/ehjcr/ytaf299)
Supplement: ytaf299_Supplementary_Data [file ytaf299_supplementary_data.zip › Supplementary Table 1.docx]

| \| **Procedural Stage and Diagnostic Modality** \| \| --- \|  \|  \| \| --- \| | \| **Prevention** \| \| --- \|  \|  \| \| --- \| | **Recognition** |
| --- | --- | --- | --- | --- | --- | --- |
| Intra-procedural Vascular Access | \| Ultrasound-guided or fluoroscopy-guided venous access; confirm venous cannulation. \| \| --- \| | \| Arterial pulsation, bright red pulsatile blood return; unusual resistance during advancement of wire or sheath. \| \| --- \| |
| Intra-procedural Fluoroscopy | Utilize orthogonal (AP, RAO, LAO) fluoroscopic views; verify venous placement by advancing guidewire below diaphragm into IVC; confirm correct RV trajectory (right side of spine, RV apex/septum). | \| Lead trajectory toward left side of spine or "knuckling" against aortic root; posterior positioning near spine (LAO); lateral trajectory suggesting coronary sinus or LV placement (RAO). \| \| --- \| |
| Intra-procedural ECG | \| Confirm expected paced QRS morphology consistent with RV placement (LBBB-like morphology). \| \| --- \| | \| Atypical paced QRS morphology, notably RBBB pattern or unusual configuration raising suspicion of LV placement. \| \| --- \| |
| Post-procedural ECG | \| Confirm standard RV pacing morphology; routinely assess immediately post-procedure. \| \| --- \| | \| Persistent RBBB-like paced pattern, inconsistent with RV placement; unexplained change in paced morphology from expected pattern. \| \| --- \| |
| Post-procedural CXR | \| Obtain routine AP and lateral CXR post-procedure; confirm leads cross midline to the right heart structures. \| \| --- \| | \| Leads positioned posteriorly or crossing spine midline in AP view; posterior lead position in lateral view suggesting LV placement or coronary sinus involvement. \| \| --- \| |
| Post-procedural Echocardiography | \| Use early echocardiography to confirm lead placement in RV chamber, particularly if imaging available intra-procedure. \| \| --- \| | \| Direct visualization of lead crossing aortic valve, located in LV; difficulty visualizing leads in RV; atypical course suggesting misplacement. \| \| --- \| |
| Post-procedural Angiography | \| If angiography is performed, verify lead positioning under fluoroscopy and consider contrast injection to clarify its trajectory. \| \| --- \| | \| Angiographic confirmation of arterial lead course; trajectory through aorta or coronary arteries. \| \| --- \| |
| Post-procedural CT | \| Use CT for cases with high suspicion or ambiguous imaging to confirm venous system placement if uncertain. \| \| --- \| | \| Definitive identification of lead trajectory through subclavian artery, aorta, or into LV; clear anatomical delineation of arterial course and final LV lead position. \| \| --- \| |
